# Analysis of HETEs in human whole blood by chiral UHPLC-ECAPCI/HRMS

**DOI:** 10.1194/jlr.D081414

**Published:** 2018-01-04

**Authors:** Liudmila L. Mazaleuskaya, Ashkan Salamatipour, Dimitra Sarantopoulou, Liwei Weng, Garret A. FitzGerald, Ian A. Blair, Clementina Mesaros

**Affiliations:** Institute for Translational Medicine and Therapeutics* University of Pennsylvania School of Medicine, Philadelphia, PA 19104-6160; Penn Superfund Research Program (SRP) Centers for Cancer Pharmacology and Excellence in Environmental Toxicology,† University of Pennsylvania School of Medicine, Philadelphia, PA 19104-6160

**Keywords:** chiral hydroxyeicosatetraenoic acids, human blood, plasma lipidomics, serum lipidomics, coagulation, hydroxyeicosatetraenoic acids, ultra-high-performance liquid chromatography-electron capture atmospheric pressure chemical ionization high-resolution mass spectrometry

## Abstract

The biosynthesis of eicosanoids occurs enzymatically via lipoxygenases, cyclooxygenases, and cytochrome P450, or through nonenzymatic free radical reactions. The enzymatic routes are highly enantiospecific. Chiral separation and high-sensitivity detection methods are required to differentiate and quantify enantioselective HETEs in complex biological fluids. We report here a targeted chiral lipidomics analysis of human blood using ultra-HPLC-electron capture (EC) atmospheric pressure chemical ionization/high-resolution MS. Monitoring the high-resolution ions formed by the fragmentation of pentafluorobenzyl derivatives of oxidized lipids during the dissociative EC, followed by in-trap fragmentation, increased sensitivity by an order of magnitude when compared with the unit resolution MS. The 12(*S*)-HETE, 12(*S*)-hydroxy-(5Z,8E,10E)-heptadecatrienoic acid [12(*S*)-HHT], and 15(*S*)-HETE were the major hydroxylated nonesterified chiral lipids in serum. Stimulation of whole blood with zymosan and lipopolysaccharide (LPS) resulted in stimulus- and time-dependent effects. An acute exposure to zymosan induced ∼80% of the chiral plasma lipids, including 12(*S*)-HHT, 5(*S*)-HETE, 15(*R*)-HETE, and 15(*S*)-HETE, while a maximum response to LPS was achieved after a long-term stimulation. The reported method allows for a rapid quantification with high sensitivity and specificity of enantiospecific responses to in vitro stimulation or coagulation of human blood.

High-resolution MS (HRMS)-based lipidomics methodology is revolutionizing the field of serum or plasma biomarker analysis. The ability to resolve enantiomeric, regioisomeric, and stereoisomeric lipids is necessary to distinguish between different pathways of formation. Bioactive lipids are formed enzymatically by cyclooxygenase (COX), lipoxygenase (LOX), and cytochrome P450, as well as nonenzymatically from reactive oxygen species (**Scheme 1**). HETEs are biologically active monohydroxy fatty acids originating from various cell types (1, 2). LOX and COX enzymes stereospecifically insert molecular oxygen into polyunsaturated fatty acids, like arachidonate, to form an intermediate product, hydroperoxyeicosatetraenoic acid, which is ultimately reduced by peroxidases to a specific HETE. The hydroxyl group of HETEs formed by LOXs is in the *S*-configuration, for most cases, while the HETEs formed by COXs have it in the *R*-configuration (2, 3). Cytochrome P450 enzymes are also capable of synthesizing HETEs with the predominant formation of the *R* enantiomer (4). Besides the enzymatic route of biosynthesis, oxidized lipids can be formed nonenzymatically through the interaction of arachidonic acid with reactive oxygen species (5). Ultimately, complex biological fluids, like whole blood, contain a racemic mixture of various oxidized lipids, which require chiral separation and a high-sensitivity method for distinction and quantification. For example, 15(*S*)-HETE is an important metabolite of 15-LOX type 1 [15-LOX-1 (mouse ortholog 12/15-LOX)], 15-LOX type 2, and of both COX isoforms, whereas its racemic stereoisomer, 15-HETE, is formed nonenzymatically during lipid peroxidation, and its enantiomer, 15(*R*)-HETE, is formed by COX enzymes (2). To enhance sensitivity, we used atmospheric pressure chemical ionization (APCI)-MS after derivatization with an electron-capturing group such as pentafluorobenzenzyl (PFB). This technique is called electron capture (EC)APCI-MS. The derivatization of bioactive lipids with PFB-bromide (PFB-Br) before LC-MS analysis is rapid and does not require further purification. We have now implemented ECAPCI-MS on the Q-Exactive HF Hybrid Quadrupole-Orbitrap mass spectrometer using chiral ultra-HPLC (UHPLC). We have demonstrated that a wide variety of HETEs can be detected in serum and plasma by chiral UHPLC-HRMS and that enantiomers, such as 15(*S*)- and 15(*R*)-HETE, can be readily separated (6). Furthermore, using UHPLC-HRMS in the ECAPCI mode, chromatography is relatively short and robust, allowing for a large number of enantiomeric pairs to be separated. The method can easily be adapted to include the enantiomeric analysis of other chiral lipids.

Scheme 1.Eicosanoid formation in humans. HPETE, hydroperoxyeicosatetraenoic acid.
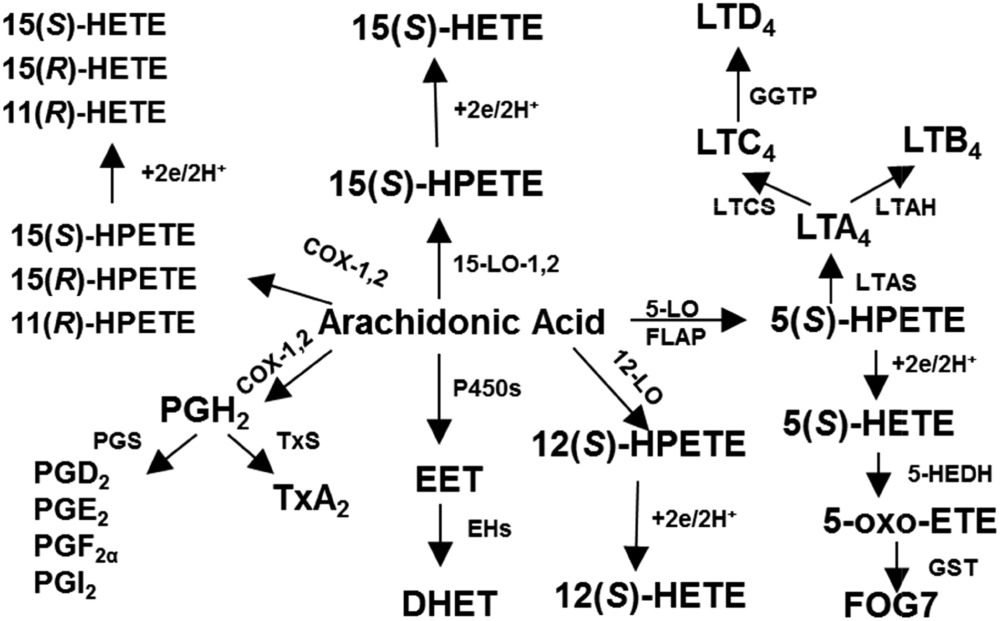


## MATERIALS AND METHODS

### Materials

Diisopropylethylamine, PFB-Br, and solvents were purchased from Sigma-Aldrich (St. Louis, MO). HPLC-grade hexane, acetonitrile, ethyl acetate, acetic acid, formic acid, isopropanol, and ethanol were obtained from Fisher Scientific Co. (Fair Lawn, NJ). ACS-grade ethanol was obtained from Pharmco (Brookfield, CT). Gases were supplied by BOC Gases (Lebanon, NJ). The Chiralpak AD-H column was obtained from Chiral Technologies (West Chester, PA). Deuterated analogs of thromboxane (Tx)B_2_ (the hydrolysis product of TxA_2_, [d_4_]-TxB_2_) and 15(*S*)-HETE ([^2^H_8_]-15(*S*)-HETE), and authentic standards for (±)5-HETE, (±)9-HETE, 11(*S*)-HETE, 11(*R*)-HETE, (±)12-HETE, 12(*S*)-hydroxy-(5Z,8E,10E)-heptadecatrienoic acid [12(*S*)-HHT], (±)15-HETE, and 20-HETE were purchased from Cayman Chemical (Ann Arbor, MI). Stock solutions were prepared in acetonitrile and stored in glass vials at −80°C. We utilized purified deionized water (Milli-Q water purification system; EMD Millipore, Billerica, MA) in the preparation of all aqueous solutions and mobile phases. Phree cartridges for phospholipid and protein removal (8B-S133-TAK) were obtained from Phenomenex (Torrance, CA). Bacterial lipopolysaccharide (LPS) from *Escherichia coli* 0111:B4 (L2630) and zymosan A from *Saccharomyces cerevisiae* (Z4250) were purchased from Sigma-Aldrich. Heparin sodium (1,000 U/ml) was purchased from Patterson Veterinary (Columbus, OH). PBS, Fisher brand disposable borosilicate glass tubes (5 ml, 75 × 12 mm, #14-961-26), Sarstedt polypropylene tubes (5 ml, 75 × 12 mm, #50-809-202), and 96-well deep sterile polypropylene plates (#260251) were purchased from Thermo Fisher Scientific (Waltham, MA). MicroClime-Environmental lids (#LLS-0310) were obtained from Labcyte (San Jose, CA).

### Sources of human plasma and serum

The clinical study was conducted in accordance with the Declaration of Helsinki. The clinical protocol (NCT02095288) was approved by the Institutional Review Board of the University of Pennsylvania and by the Advisory Council of the Center for Human Phenomic Science of the University of Pennsylvania. All the participants provided written informed consent, and were healthy, nonsmoking, and nonpregnant volunteers. The participants refrained from all the medications, including NSAIDs, for at least 2 weeks before a blood donation. Human whole blood was drawn by venipuncture. For plasma collection, whole blood was drawn with syringes containing 10 IU of sodium heparin and distributed in 500 μl aliquots into 96-well deep sterile polypropylene plates. Zymosan (125 μg/ml final concentration) or LPS (100 μg/ml final concentration) was added to the blood in 20 μl aliquots of PBS for single agonist stimulation or in 10 μl aliquots each for a combination of stimuli, and incubated for 4 and 24 h at 37°C. Plates were covered with MicroClime-Environmental lids to minimize edge effects. After the incubation, blood was spun down at 3,000 *g* for 10 min at 4°C and plasma was removed for ECAPCI/HRMS analysis. Blood samples, spun down in polypropylene tubes immediately after the blood draw, served as untreated controls. Whole blood from fifteen human volunteers was used for in vitro stimulation with LPS or zymosan (n = 15). Coincubation of LPS and zymosan was performed in whole blood from five independent subjects (n = 5). For serum preparation, 500 μl aliquots of human whole blood were incubated in glass tubes at 37°C for 1 h and serum was isolated for UHPLC-ECAPCI/HRMS analysis. Nine genetically unrelated human volunteers provided blood for serum collection (n = 9).

### Chiral eicosanoid extraction and synthesis of PFB derivatives

Plasma and serum samples (200 μl) were spiked with 1 ng of the stable isotope-labeled internal standard for 15(*S*)-HETE ([^2^H_8_]-15(*S*)-HETE) in 900 μl of acetonitrile. Then, plasma and sera were incubated with 1% formic acid at room temperature for 15 min. After that, samples were sonicated for 1 min and the supernatants were transferred to Phree cartridges for phospholipid and protein removal. The samples were eluted with a slight vacuum (<20 kPa) and dried under a gentle stream of nitrogen at the ambient temperature. The PFB derivatives were prepared by dissolving the residues from extracted plasma and sera in 100 μl of diisopropylethylamine in acetonitrile (1:19, v/v) followed by the addition of 50 μl of PFB-Br in acetonitrile (1:9, v/v) and the incubation of the solution at 60°C for 30 min. The solution was evaporated to dryness under a nitrogen stream at room temperature and redissolved in 100 μl of hexane/ethanol (97:3, v/v). A 5 μl aliquot of each sample was injected for chiral UHPLC-HRMS analysis.

### Chiral UHPLC-ECAPCI/HRMS

Normal-phase chiral chromatography was performed using an UltiMate 3000 binary UPLC equipped with a refrigerated autosampler (6°C) and a column heater (35°C). Gradient elution was performed in the linear mode with some modification of a previously described method (7). A Chiralpak AD-H column (250 × 4.6 mm i.d., 5 μm; Daicel Chemical Industries, Ltd., Tokyo, Japan) was employed with a flow rate of 1 ml/min. Solvent A was hexanes and solvent B was 2-propanol/methanol (5/5, v/v). The linear gradient was as follows: 2% B at 0 min, 2% B at 3 min, 8% B at 11 min, 8% B at 13 min, 50% B at 14 min, 50% B at 18 min, and 2% B at 18.5 min with an equilibration step for the next 2.5 min. The column effluent was diverted to waste before 3 min and after 13 min. MS was conducted on a Thermo QExactive HF HRMS. The mass spectrometer was equipped with an APCI source operating in negative electron capturing ion mode. The operating conditions were as follows: vaporizer temperature, 450°C; heated capillary temperature, 320°C; and corona discharge needle, set at 30 μA. The sheath gas (nitrogen) and auxiliary gas (nitrogen) pressures were 40 psi and 10 (arbitrary units), respectively. The S lens was 60. The QE HF was alternating between full scan (*m/z* 100–600) at a resolution of 30,000 and parallel reaction monitoring (PRM) at 120,000 resolutions with a precursor isolation window of *m/z* 2 with normalized collision energy 20. The molecular [M^−^] precursor was 319.23 for all the HETEs and 327.27 for [^2^H_8_]-15(*S*)-HETE that was used as an internal standard. Quantification was done based on the most intense product ion for each of the HETEs with ±1.5 ppm. The analysis of serum TxB_2_ was performed as previously described (6).

### Chiral data analysis

All data analyses were performed using Xcalibur software version 2.0 SR2 (Thermo Electron Corporation) from raw mass spectral data. Calibration standard samples were prepared with charcoal-stripped FBS. Calibration samples were spiked with the authentic standards of (±)5-HETE, (±)9-HETE, 11(*S*)-HETE, 11(*R*)-HETE, (±)12-HETE, 12(*S*)-HHT, (±)15-HETE, and 20-HETE in the amounts of 0, 0.1, 0.25, 0.5, 1, 2.5, 5, and 10 ng, and 1 ng of the internal standard [^2^H_8_]-15(*S*)-HETE. Lipids were extracted, purified, derivatized, and analyzed as described above for the analytical samples. Calibration curves were plotted using a linear regression of the peak area ratio of analytes against the internal standard. Concentrations of the chiral products were calculated by interpolation from the calculated regression lines. Data were normalized to sample volume and expressed as lipid concentration in nanograms per milliliter.

### Statistics

Statistical analyses were performed using GraphPad Prism software version 5.0 for Mac OS X. Data represent the mean ± SEM of 9 donors for serum and plasma from untreated blood and of 5–15 donors for plasma from stimulated blood. The statistical significance of the differences between various groups was sought by unpaired two-tailed *t*-tests for comparison of serum and untreated plasma, and by paired two-tailed *t*-tests for the enantiomers in serum. To analyze the differences between stimulation groups in plasma per analyte, one-way ANOVA was used followed by Dunn’s multiple comparison test. To compare the means of lipids between serum and plasma for all analytes per donor, two-way ANOVA was used. To analyze the differences between stimulation conditions for all analytes per time point, two-way ANOVA was used followed by Dunn’s test with Bonferroni *P*-value adjustment. *P* < 0.05 was considered statistically significant. Plots with lipids expressed in nanograms per milliliter were built using GraphPad Prism software. Principal component analysis (PCA) and visualizations were performed using custom R scripts.

## RESULTS

### HR-MS/MS analysis of eicosanoids-PFB by ECAPCI

**Fig. 1.**
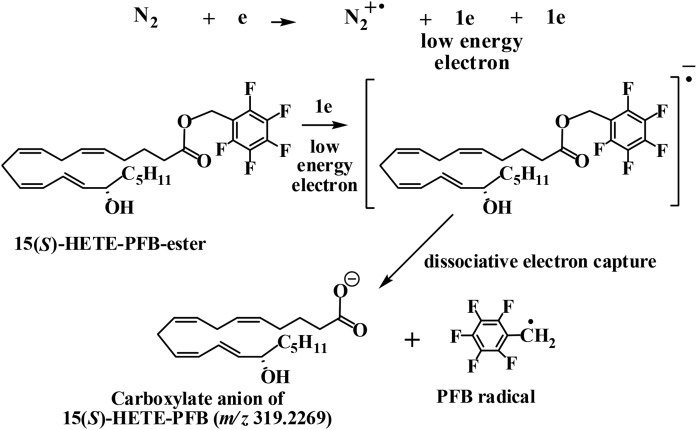
ECAPCI-MS/HRMS of 15-HETE-PFB. The low energy electrons generated from corona discharge interact with the nitrogen sheath gas and generate radical cations. Dissociative EC results in a very strong product ion corresponding to 15-HETE anion *m/z* 319.2269.

**Fig. 2.**
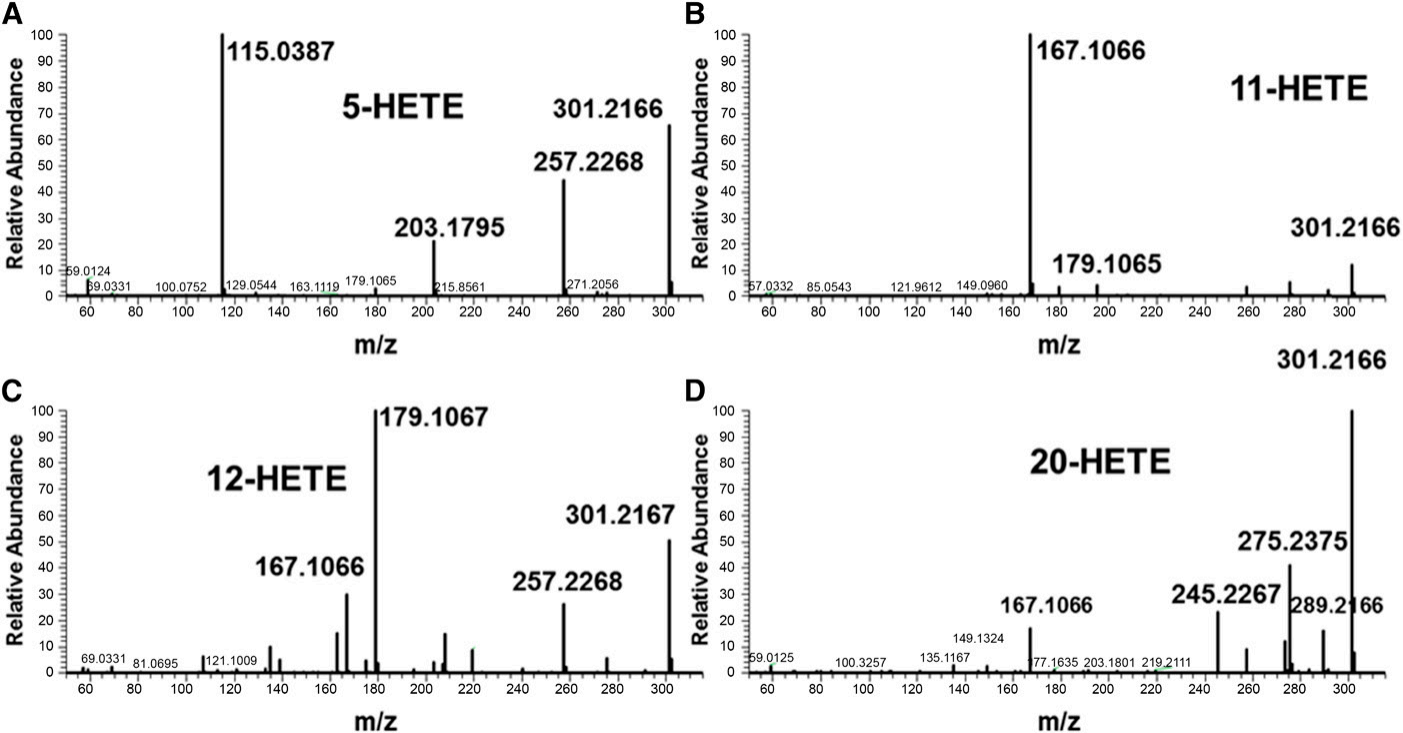
Product spectra from ECAPCI-MS/HRMS of different HETE-PFBs. Collision-induced dissociation of the parent [HETE-PFB]^−^ generates specific product ions for each stereoisomer. 5-HETE (A), 11-HETE (B), 12-HETE (C), and 20-HETE (D).

PFB derivatives of eicosanoids were analyzed after chromatographic separation under negative mode using APCI. All HETE-PFBs exhibited an intense ion at *m/z* 319.2269 (C_20_H_31_O_3_^−^ 3.1 ppm) that corresponded to the dissociative EC that occurred for all HETE-PFBs, as shown in **Fig. 1** for 5(*S*)-HETE-PFB. Collision-induced dissociation and PRM analysis were performed on the [HETE-PFB]^−^ ion *m/z* 319.22. All HETE isomers had the same molecular ion, so only one PRM was used to determine the HRMS/MS data. **Figure 2** shows examples of the HRMS/MS spectra for some HETEs. The most intense product ions for each PFB derivative are listed in **Table 1**. All the HETEs and 12(*S*)-HHT showed MS/HRMS like those reported from low-resolution instruments (data not shown) (8–12).

**TABLE 1.** Selected product ions for chiral analysis by UHPLC-ECAPCI-MS/HRMS and corresponding retention times

Analyte	Selected Product Ion (*m/z* ± 1.5 ppm)	Retention Time (min)
5(*R*)-HETE	115.0387	11.0
5(*S*)-HETE	115.0387	11.3
8(*R*)-HETE	155.0701	9.3
8(*S*)-HETE	155.0701	9.9
9(*R*)-HETE	167.0704	9.4
9(*S*)-HETE	167.0704	9.6
11(*R*)-HETE	167.1066	8.9
11(*S*)-HETE	167.1066	9.8
12(*R*)-HETE	179.1067	9.4
12(*S*)-HETE	179.1067	9.7
15(*R*)-HETE	219.1386	9.6
15(*S*)-HETE	219.1386	11.1
12(*S*)-HHT	279.1966	11.4
20-HETE	245.2267	9.9

### Chiral separation of eicosanoids-PFB

Characteristic HRMS product ions were selected for each of the HETE isomers based on the most intense product ion. The chiral separation was modified from a previous report (7), shortening the gradient to reduce the time for analysis. Switching to the UPLC system, the enantiomers for 5-HETE were baseline separated with 0.3 min between 5(*R*)-HETE (11.0 min) and 5(*S*)-HETE (11.3 min). This separation is challenging to achieve with a normal HPLC system, but on the UPLC system, the retention times exhibit less than 0.1 min shifts after running more than 300 samples.

**Fig. 3.**
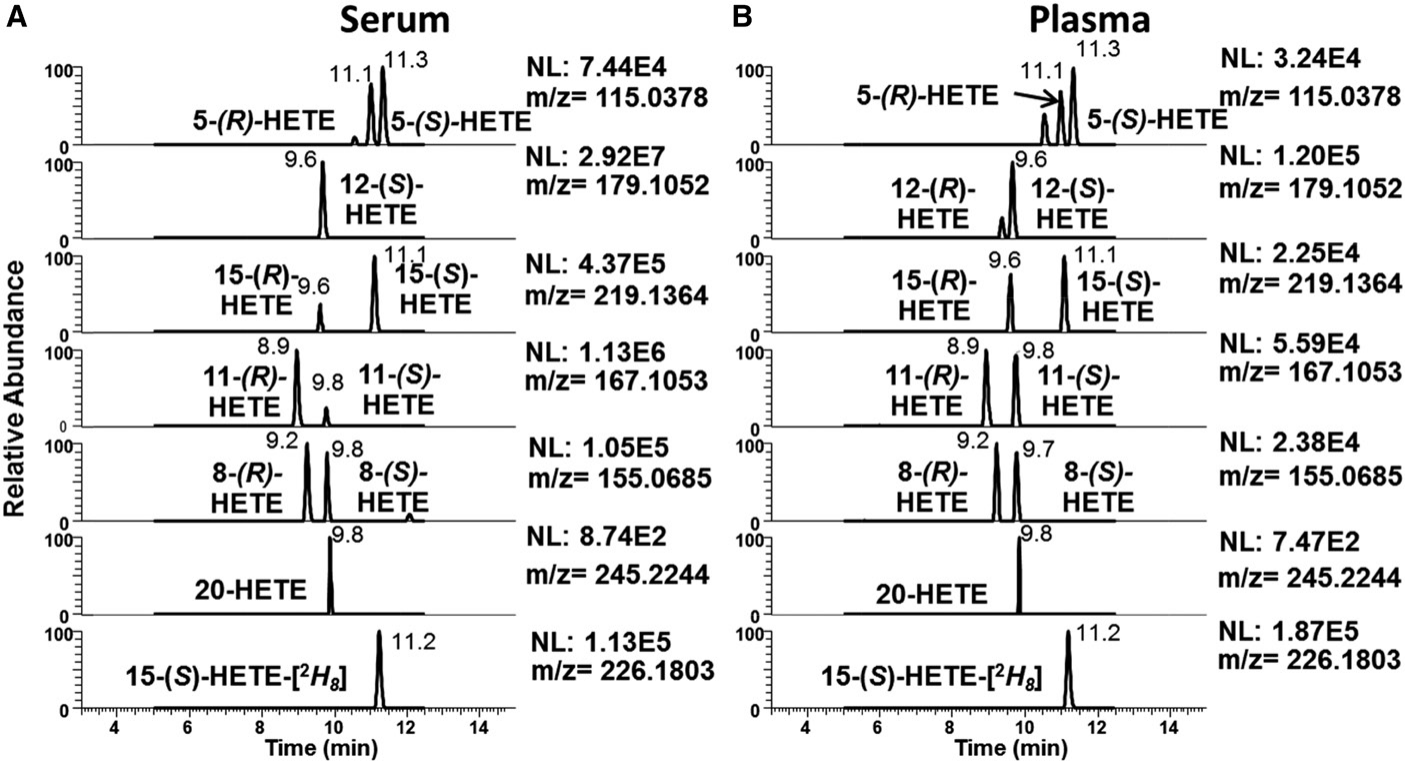
Typical LC-ECAPCI-MS/HRMS chromatograms of HETEs as PFB derivatives. A: Extracted from 0.2 ml human serum and spiked with a synthetic heavy isotope analog internal standard {1 ng of [^2^H_8_]-15(*S*)-HETE}. B: Extracted from 0.2 ml human plasma and spiked with a synthetic heavy isotope analog internal standard {1 ng of [^2^H_8_]-15(*S*)-HETE}.

The chiral LC-ECAPCI-MS/HRMS profile of human serum and plasma from unstimulated whole blood (**Fig. 3**) identified most of the chiral HETEs, but due to the high concentration of 12(*S*)-HETE, 12(*R*)-HETE was only detected in plasma, but not in serum. The chromatogram showed that 15(*R*)-HETE [retention time (rt) 9.6 min] was present in a 1:3 ratio to 15(*S*)-HETE (rt 11.1 min) in serum, but the ratio was closer to 1:1 in human plasma. The 11(*R*)-HETE (rt 8.9 min) was the major enantiomer present in serum, but in the plasma the enantiomers exhibited less enantiospecificity with the 11(*S*)-HETE (rt 9.8 min), showing about the same intensity. The 8(*R*)-HETE (rt 9.2 mi) and 8(*S*)-HETE (rt 9.8 min) showed no enantioselectivity in serum or plasma, and the same was true about 9(*R*)-HETE (rt 9.4 mi) and 9(*S*)-HETE (rt 9.6 min). Both serum and plasma from unstimulated whole blood showed 20-HETE (rt 9.8 min).

Quantitation was performed based on the MS/HRMS data using a 3 ppm (±1.5 ppm) window with qualifying peaks from the HR full scan data. Reinjection of samples after storage in the autosampler for 24 and 48 h resulted in the calculated amounts of the eicosanoids and signal intensity within a 5% coefficient of variation (data not shown).

### Chiral eicosanoid formation during coagulation of human whole blood

**Fig. 4.**
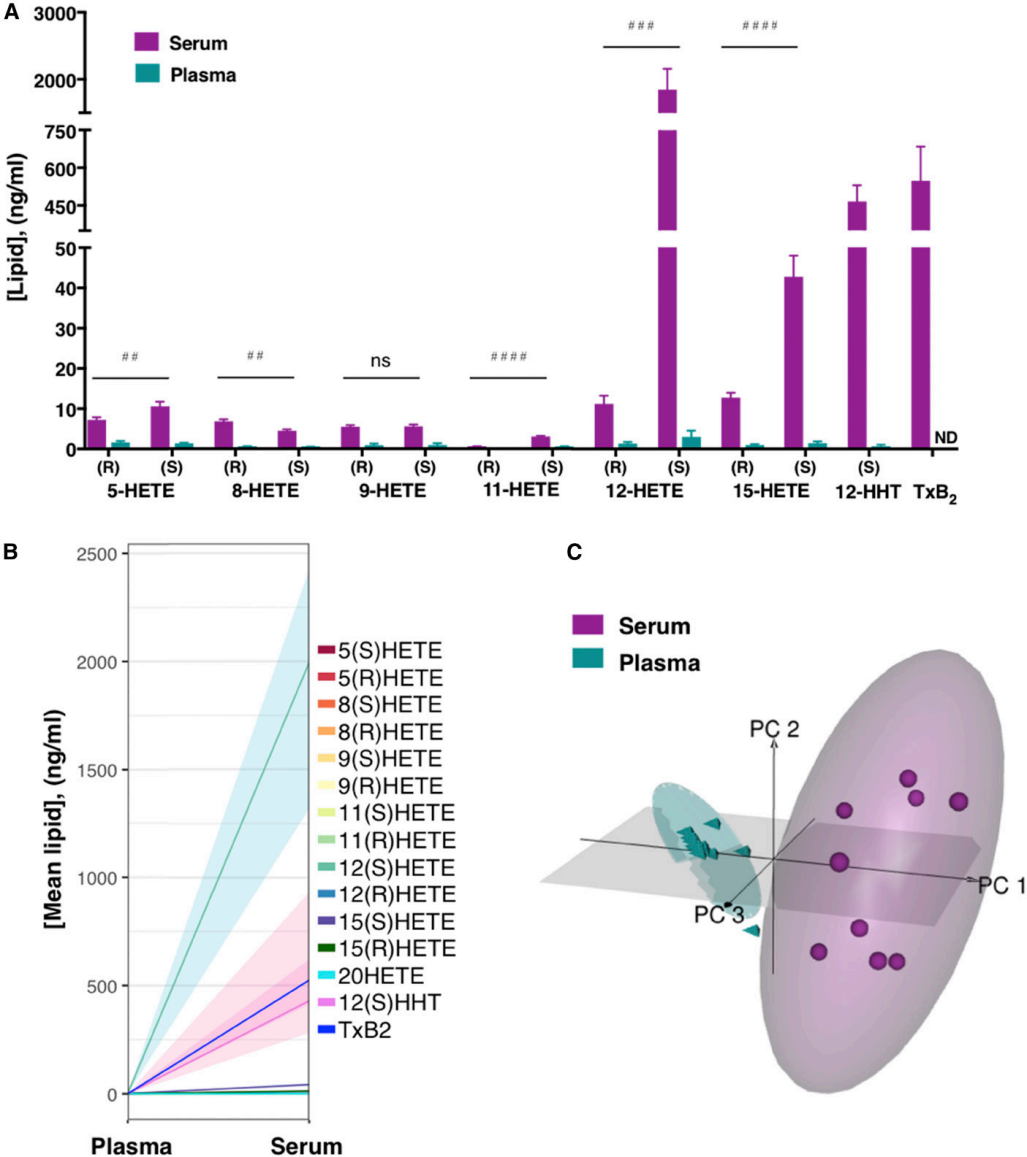
Enantioselective formation of HETEs and Tx during the intrinsic pathway of coagulation of human blood. Human whole blood was incubated at 37°C for 1 h and serum was removed for analysis of TxB_2_ and HETEs, as described in the Materials and Methods. Lipids from serum were compared with plasma lipids from untreated whole blood. A, B: Data are expressed as nanograms of lipid per sample volume and represent the mean ± SEM; ^##^*P* ≤ 0.01, ^###^*P* ≤ 0.001, ^####^*P* ≤ 0.0001 between enantiomers for serum; paired *t*-test; n = 9. ND, not detected; ns, not significant. Shaded areas depict interquartile range of distribution of the lipid concentrations. Absolute quantities of lipids are summarized in Table 1. C: PCA of serum and plasma lipids presented in a three-dimensional loading plot. Each symbol represents an independent subject (n = 9). Ellipses denote 95% confidence regions. Two-way ANOVA of the means of serum versus plasma for all analytes, *P* = 2 × 10^−16^.

The UHPLC-ECAPCI/HRMS method was first applied to the analysis of chiral HETEs in coagulated whole blood (**Fig. 4**, **Table 2**). The 12(*S*)-HETE was the most abundant hydroxylated lipid in serum, averaging at 1,849 ± 308 ng/ml (mean ± SEM, n = 9) (Fig. 4A, B). The 12(*S*)-HHT was the second most abundant chiral species (466.8 ± 64.6 ng/ml), which was comparable in its amount to TxB_2_ (548.9 ± 136.6 ng/ml). Finally, 15(*S*)-HETE was the third most abundant chiral HETE averaging at 42.75 ± 5.2 ng/ml. A striking difference between enzymatic and nonenzymatic products was observed for the enantiomers of 12-HETE in serum (Fig. 4A). The levels of the enzymatically made 12(*S*)-HETE were markedly higher than those of the nonenzymatically formed 12(*R*)-HETE (1,849 ± 308 ng/ml vs. 11.16 ± 2 ng/ml, respectively, *P* ≤ 0.001). There was also somewhat more 15(*S*)-HETE than 15(*R*)-HETE (42.75 ± 5.2 ng/ml vs. 12.74 ± 1.2 ng/ml, respectively, *P* ≤ 0.0001). Finally, despite low absolute values, chiral products of 11-HETE showed an almost 6-fold difference in the enantiomer formation in serum (3.05 ± 0.2 ng/ml 11(*S*)-HETE vs. 0.54 ± 0.1 ng/ml 11(*R*)-HETE, *P* ≤ 0.0001). Modest, although significant, differences were detected in the levels of 5-HETE and 8-HETE enantiomers in serum (Fig. 4A). The 5(*S*)-HETE prevailed over the *R*-enantiomer (10.59 ± 1.2 ng/ml vs. 7.26 ± 0.6 ng/ml, respectively, *P* ≤ 0.01), as did 8(*R*)-HETE over 8(*S*)-HETE (6.86 ± 0.5 ng/ml vs. 4.44 ± 0.4 ng/ml; *P* ≤ 0.01). Finally, blood clotting led to nonenzymatic release of low, but detectable, levels of 9-HETE enantiomers (5.48 ± 0.4 ng/ml of 9(*R*)-HETE and 5.58 ± 0.5 ng/ml of 9(*S*)-HETE) and 11(*S*)-HETE (3.05 ± 0.2 ng/ml). PCA demonstrated a clear separation between the serum and plasma lipidomes based on 15 different analytes collected from nine distinct individuals (n = 9) (Fig. 4C). Most (97%) of the variation was explained with three principal components. Principal component 1 explained 86% of the variance and correlated similarly with all the analytes. The ellipses in the plot denote 95% confidence areas. A statistically significant difference was detected between serum versus plasma (*P* = 2 × 10^−16^). The 20-HETE was the only analyte that remained low during coagulation and did not significantly change from the untreated plasma levels (0.15 ± 0.05 ng/ml vs. 0.06 ± 0.03 ng/ml, respectively) (Table 2). Plasma from untreated blood contained measurable levels of all the HETEs studied and 12(*S*)-HHT, while TxB_2_ was not detected (Fig. 4A, B; Table 2). Among the chiral enantiomers in untreated plasma, 11(*S*)-HETE levels were much higher than those of 11(*R*)-HETE (0.49 ± 0.2 ng/ml vs. 0.02 ± 0.01 ng/ml; *P* ≤ 0.05).

**TABLE 2.** Levels of HETEs and TxB_2_ in human serum versus plasma from untreated whole blood determined by LC-ECAPCI-MS/HRMS

Analyte	Plasma (mean ± SEM, ng/ml)	Serum (Mean ± SEM, ng/ml)	*P* value (Plasma vs. Serum)
5(*R*)-HETE	1.64 ± 0.3	7.26 ± 0.6*^a^*	≤0.0001
5(*S*)-HETE	1.32 ± 0.2	10.59 ± 1.2	≤0.0001
8(*R*)-HETE	0.58 ± 0.1	6.86 ± 0.5*^a^*	≤0.0001
8(*S*)-HETE	0.48 ± 0.1	4.44 ± 0.4	≤0.0001
9(*R*)-HETE	0.92 ± 0.4	5.48 ± 0.4^ns^	≤0.0001
9(*S*)-HETE	0.95 ± 0.4	5.58 ± 0.5	≤0.0001
11(*R*)-HETE	0.02 ± 0.01*^b^*	0.54 ± 0.1*^c^*	≤0.0001
11(*S*)-HETE	0.49 ± 0.2	3.05 ± 0.2	≤0.0001
12(*R*)-HETE	1.33 ± 0.4	11.16 ± 2*^d^*	≤0.001
12(*S*)-HETE	2.99 ± 1.5	1,848.9 ± 308	≤0.0001
15(*S*)-HETE	0.96 ± 0.3	12.74 ± 1.2*^c^*	≤0.0001
15(*S*)-HETE	1.46 ± 0.4	42.75 ± 5.2	≤0.0001
12(*S*)-HHT	0.6 ± 0.4	466.82 ± 64.6	≤0.0001
20-HETE	0.06 ± 0.03	0.15 ± 0.05	ns
TxB_2_	ND	548.93 ± 136.6	≤0.001

Human whole blood was incubated at 37°C for 1 h and serum was removed for analysis of TxB_2_ and chiral HETEs, as described in the Materials and Methods. Lipids from serum were compared with plasma lipids from untreated whole blood. Data are expressed as mean ± SEM, serum versus plasma, unpaired two-tailed *t*-test, n = 9. ND, not detected; ns, not significant.

a *P* ≤ 0.01, (*R*) versus (*S*) enantiomers, paired two-tailed *t*-test, n = 9.

b *P* ≤ 0.05, (*R*) versus (*S*) enantiomers, paired two-tailed *t*-test, n = 9.

c *P* ≤ 0.0001, (*R*) versus (*S*) enantiomers, paired two-tailed *t*-test, n = 9.

d *P* ≤ 0.001, (*R*) versus (*S*) enantiomers, paired two-tailed *t*-test, n = 9.

### Chiral eicosanoids in plasma from stimulated human whole blood

**Fig. 5.**
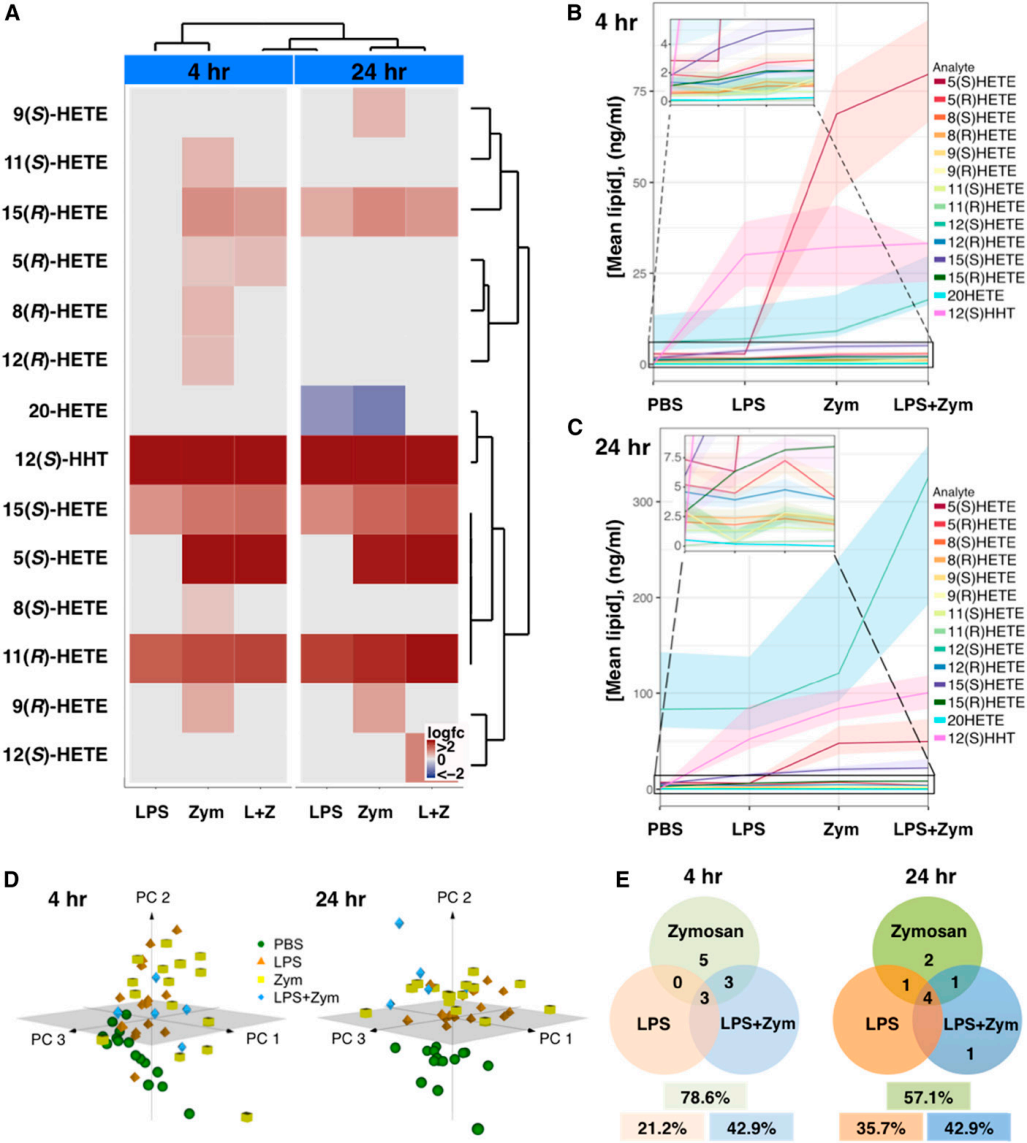
Time- and stimulus-dependent effects of in vitro stimulation on chiral HETEs in human whole blood. Heparinized human whole blood was stimulated with 100 μg/ml LPS and 125 μg/ml zymosan (Zym), alone (n = 15) or in combination (L+Z, n = 5) for 4 and 24 h at 37°C. Plasma was removed for analysis of chiral HETEs as described in the Materials and methods, and significant fold differences over the vehicle (PBS) control were summarized on a log scale in a heat map (A). Red and blue boxes denote elevation and reduction, respectively. Unpaired two-tailed *t*-test, n = 5–15. Absolute quantities of lipids are summarized in Table 2. Effects of stimulating conditions on lipid production at 4 h (B) and 24 h (C). Shaded areas depict interquartile range of distribution of the lipid concentrations. D: PCA of plasma lipids after stimulation of whole blood for 4 h and 24 h and presented in three-dimentional loading plots. Each symbol represents an independent subject (n = 5–15). E: Venn diagrams showing the number of analytes common or unique to treatments after 4 h and 24 h of stimulation, relative to PBS control. The percentage of total analytes affected by a specific treatment is shown in boxes.

**Fig. 6.**
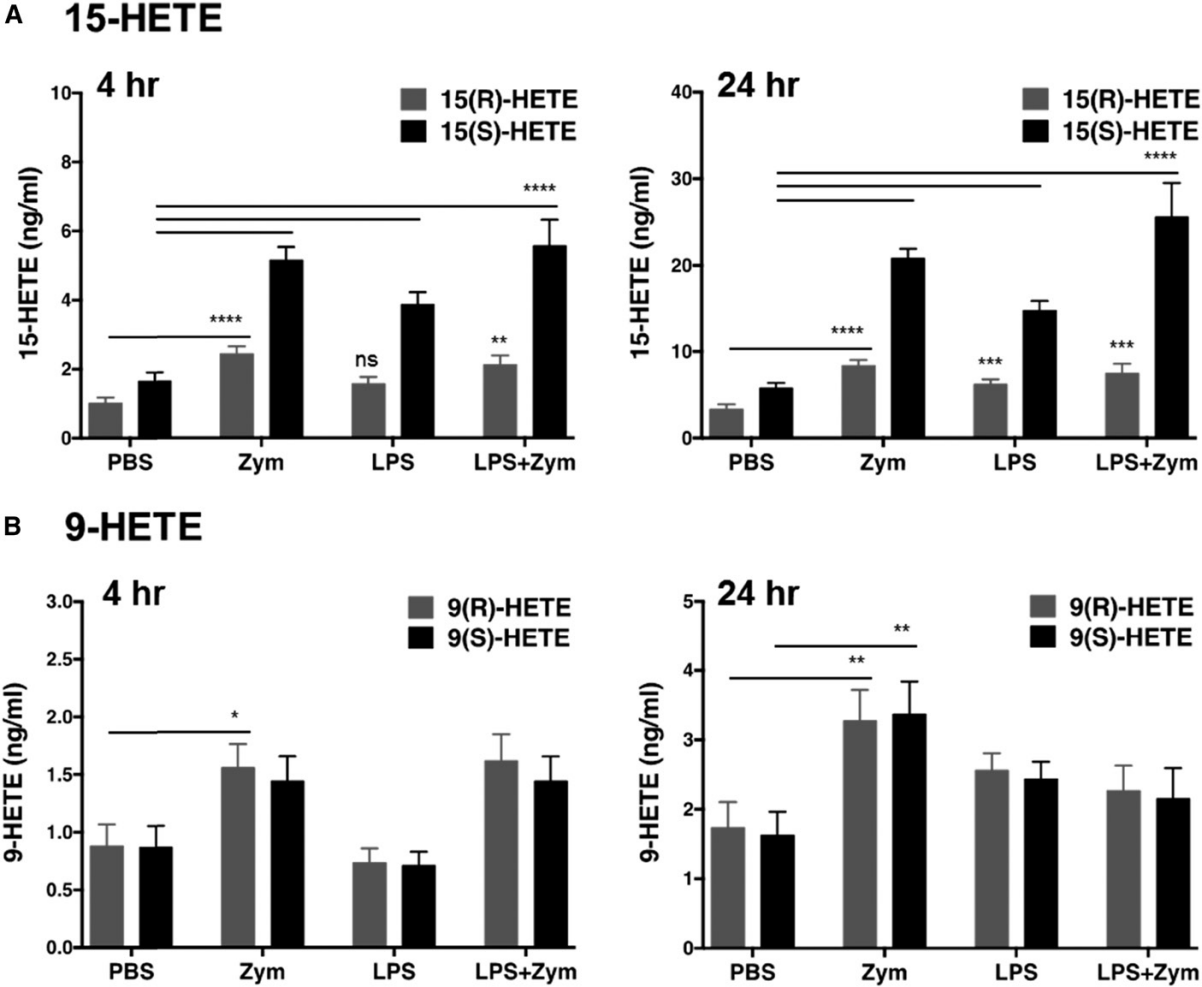
Enantioselective biosynthesis of 15-HETE compared with nonenzymatic formation of 9-HETE in stimulated human blood. Heparinized human whole blood was stimulated with 100 μg/ml LPS and 125 μg/ml zymosan (Zym), alone (n = 15) or in combination (LPS+Zym, n = 5), for 4 and 24 h at 37°C. Plasma was removed for analysis of 15-HETE (A) and 9-HETE (B) as described in the Materials and Methods. Data are expressed as mean ± SEM; *P ≤ 0.05, **P ≤ 0.01, ***P ≤ 0.001, ****P ≤ 0.0001 versus PBS; one-way ANOVA, Dunnett’s test; n = 5–15. ns, not significant.

Stimulation of human whole blood with LPS and zymosan, alone or in combination, resulted in stimulus- and time-dependent effects on chiral HETEs (**Fig. 5**, **Table 3**). At 4 h and 24 h, LPS triggered increasing production of 12(*S*)-HHT (30.9 ± 2.8 ng/ml vs. 61.44 ± 7 ng/ml, mean ± SEM, n = 15), 15(*S*)-HETE (3.9 ± 0.3 ng/ml vs. 14.9 ± 1 ng/ml), and 11(*R*)-HETE (0.09 ± 0.01 ng/ml vs. 0.32 ± 0.03 ng/ml), and modestly stimulated 15(*R*)-HETE (6.3 ± 0.5 ng/ml vs. PBS 3.41 ± 0.5 ng/ml, *P* ≤ 0.001) at 24 h postincubation (Fig. 5A–C, **Fig. 6A**). The pairwise comparison between different treatments by subject revealed significant changes in the lipidomic responses to all stimuli at 4 h (PBS vs. zymosan, *P* ≤ 0.0001; PBS vs. LPS, *P* ≤ 0.05; PBS vs. LPS + zymosan, *P* ≤ 0.0001) and for zymosan-treated blood at 24 h (*P* ≤ 0.001) (Fig. 5D). Overall, LPS affected ∼20% of the lipids studied by 4 h and ∼36% of the lipids by 24 h (Fig. 5E). In comparison, zymosan had a more global effect on the plasma lipidome, affecting ∼80% and ∼60% of the studied lipids at 4 h and 24 h, accordingly. After a short-term incubation, five analytes were uniquely upregulated by zymosan including 8(*R*)-HETE (1.36 ± 0.2 ng/ml, *P* ≤ 0.05), 8(*S*)-HETE (1.18 ± 0.2 ng/ml, *P* ≤ 0.05), 9(*R*)-HETE (1.56 ± 0.2 ng/ml, *P* ≤ 0.05), 11(*S*)-HETE (0.78 ± 0.09 ng/ml, *P* ≤ 0.05), and 12(*R*)-HETE (2.11 ± 0.2 ng/ml, *P* ≤ 0.05). However, elevations of 8-HETE enantiomers, 11(*S*)-HETE and 12(*R*)-HETE, were transient and became indistinguishable from PBS control by 24 h. At 24 h, zymosan potentiated 9(*R*)-HETE (3.28 ± 0.4 ng/ml, *P* ≤ 0.01) even more than early on and significantly upregulated the *S*-enantiomer of 9-HETE (3.38 ± 0.5 ng/ml, *P* ≤ 0.01) (Fig. 6B). Both enantiomers of 5-HETE were induced by zymosan at 4 h, with 5(*S*)-HETE predominating over 5(*R*)-HETE by more than 20-fold (70.24 ± 7.6 ng/ml vs, 2.98 ± 0.2 ng/ml, respectively). Relative to zymosan alone, costimulation with LPS did not trigger significantly different levels of 5(*S*)-HETE (86.6 ± 13.4 ng/ml, mean ± SEM, n = 5) or 5(*R*)-HETE (3.27 ± 0.4 ng/ml). At 24 h, only the *S*-enantiomer of 5-HETE was increased by zymosan, averaging at 53.13 ± 5.6 ng/ml and remaining comparably elevated in the costimulation group (55.83 ± 11 ng/ml). Unlike LPS, a 4 h stimulation with zymosan induced 15(*R*)-HETE (2.46 ± 0.2 ng/ml) that remained elevated with the addition of LPS (2.14 ± 0.3 ng/ml) (Fig. 6A). Zymosan-stimulated 15(*S*)-HETE averaged at 5.19 ± 0.4 ng/ml by 4 h, which was significantly more than LPS alone (*P* ≤ 0.05) and comparable to the levels induced by a combination of stimuli (5.6 ± 0.7 ng/ml). At 24 h, zymosan was more potent than LPS at upregulating both enantiomers of 15-HETE. The 15(*R*)-HETE in zymosan-treated blood averaged at 8.44 ± 0.6 ng/ml (*P* ≤ 0.05 vs. LPS) and was like the costimulated group (7.58 ± 1 ng/ml); while 15(*S*)-HETE averaged at 20.91 ± 1 ng/ml (*P* ≤ 0.01 vs. LPS) after zymosan and at 25.7 ± 3.8 ng/ml (*P* ≤ 0.001 vs. LPS) after the coincubation of zymosan and LPS. Like LPS, zymosan triggered 12(*S*)-HHT (32.6 ± 2.9 ng/ml) and 11(*R*)-HETE (0.1 ± 0.02 ng/ml) at 4 h; a coincubation of LPS with zymosan resulted in comparable levels of these analytes relative to the individual stimuli alone. However, at 24 h, zymosan induced 12(*S*)-HHT (89.1 ± 6.6 ng/ml) more potently than LPS (61.44 ± 7 ng/ml, *P* ≤ 0.05). The 12(S)-HHT was potentiated even more in the costimulation group (110.7 ± 20 ng/ml, *P* ≤ 0.01 vs. LPS). A prolonged incubation with zymosan resulted in comparable induction of 11(*R*)-HETE (0.38 ± 0.03 ng/ml) relative to LPS. Finally, in vitro stimulation of whole blood for 4 h had no effect on 12(*S*)-HETE and 20-HETE production. Interestingly, a prolonged exposure to LPS (0.19 ± 0.04 ng/ml, *P* ≤ 0.05 vs. PBS 0.52 ± 0.11 ng/ml) or zymosan (0.23 ± 0.06 ng/ml, *P* ≤ 0.05) had inhibitory effects on 20-HETE. After a long-term incubation, there was a trend to elevate 12(*S*)-HETE in stimulated whole blood, however, the data reached significance only in the presence of both stimuli [342.18 ± 110 ng/ml (n = 5) vs. PBS 125.93 ± 26.7 ng/ml (n = 15), *P* ≤ 0.05).

**TABLE 3.** Levels of chiral HETEs in human plasma determined by LC-ECAPCI-MS/HRMS

Analyte	4 h (Mean ± SEM, ng/ml)				24 h (Mean ± SEM, ng/ml)			
	PBS (n = 15)	LPS (n = 15)	Zymosan (n = 15)	LPS + Zymosan (n = 5)	PBS (n = 15)	LPS (n = 15)	Zymosan (n = 15)	LPS + Zymosan (n = 5)
5(*R*)-HETE	2.12 ± 0.2	1.75 ± 0.1	2.98 ± 0.2*^a^*	3.27 ± 0.4*^a^*	5.96 ± 0.7	4.98 ± 0.4	7.44 ± 0.7	4.89 ± 0.6
5(*S*)-HETE	2.75 ± 0.2	3.48 ± 0.6	70.24 ± 7.6*^b^*	86.6 ± 13.4*^b^*	7.58 ± 0.7	6.48 ± 0.54	53.13 ± 5.6*^b^*	55.83 ± 11*^b^*
8(*R*)-HETE	0.85 ± 0.2	0.68 ± 0.1	1.36 ± 0.2*^a^*	1.23 ± 0.2	2.79 ± 0.4	2.55 ± 0.2	3.16 ± 0.4	2.18 ± 0.3
8(*S*)-HETE	0.65 ± 0.1	0.62 ± 0.1	1.18 ± 0.2*^a^*	1.16 ± 0.2	2.43 ± 0.4	1.98 ± 0.2	2.67 ± 0.4	1.75 ± 0.4
9(*R*)-HETE	0.89 ± 0.2	0.74 ± 0.1	1.56 ± 0.2*^a^*	1.62 ± 0.2	1.74 ± 0.4	2.57 ± 0.2	3.28 ± 0.4*^c^*	2.28 ± 0.4
9(*S*)-HETE	0.87 ± 0.2	0.71 ± 0.1	1.44 ± 0.2	1.44 ± 0.2	1.63 ± 0.3	2.45 ± 0.2	3.38 ± 0.5*^c^*	2.16 ± 0.4
11(*R*)-HETE	0.02 ± 0.003	0.09 ± 0.01*^b^*	0.1 ± 0.01*^b^*	0.1 ± 0.02*^b^*	0.06 ± 0.008	0.32 ± 0.03*^b^*	0.38 ± 0.03*^b^*	0.46 ± 0.1*^b^*
11(*S*)-HETE	0.48 ± 0.07	0.47 ± 0.05	0.78 ± 0.09*^a^*	0.66 ± 0.08	1.6 ± 0.2	1.39 ± 0.1	1.89 ± 0.2	1.34 ± 0.2
12(*R*)-HETE	1.39 ± 0.2	1.27 ± 0.2	2.11 ± 0.2*^a^*	1.96 ± 0.3	4.14 ± 0.4	3.99 ± 0.3	5.1 ± 0.5	4.05 ± 0.2
12(*S*)-HETE	10.24 ± 2.4	14.59 ± 4.5	18.2 ± 4.8	31.22 ± 14.5	125.93 ± 26.7	132.07 ± 31.5	201.88 ± 44.9	342.18 ± 110*^a^*
15(*S*)-HETE	1.04 ± 0.1	1.59 ± 0.2	2.46 ± 0.2*^b^*	2.14 ± 0.3*^c^*	3.41 ± 0.5	6.3 ± 0.5*^d^*	8.44 ± 0.6*^b^*	7.58 ± 1*^d^*
15(*S*)-HETE	1.67 ± 0.2	3.9 ± 0.3*^b^*	5.19 ± 0.4*^b^*	5.6 ± 0.7*^b^*	5.85 ± 0.5	14.9 ± 1*^b^*	20.91 ± 1*^b^*	25.7 ± 3.8*^b^*
12(*S*)-HHT	0.22 ± 0.1	30.9 ± 2.8*^b^*	32.6 ± 2.9*^b^*	32.6 ± 5.4*^b^*	1.24 ± 0.6	61.44 ± 7*^b^*	89.1 ± 6.6*^b^*	110.7 ± 20*^b^*
20-HETE	0.16 ± 0.05	0.15 ± 0.06	0.22 ± 0.05	0.3 ± 0.14	0.52 ± 0.11	0.19 ± 0.04*^a^*	0.23 ± 0.06*^a^*	0.21 ± 0.13

Human whole blood was stimulated with 100 μg/ml LPS and 125 μg/ml zymosan, alone or in combination (LPS + Zymosan), for 4 and 24 h at 37°C. Plasma was removed for analysis of HETEs as described in the Materials and Methods. Data are expressed as mean ± SEM, one-way ANOVA, Dunn’s test, n = 5–15.

a *P* < 0.05 versus PBS at the corresponding time point.

b *P* ≤ 0.0001 versus PBS at the corresponding time point.

c *P* ≤ 0.01 versus PBS at the corresponding time point.

d *P* ≤ 0.001 versus PBS at the corresponding time point.

## DISCUSSION

The analytical method reported here employs chiral UHPLC-ECAPCI/HRMS and allows comprehensive, rapid, and highly sensitive quantification of isomeric and enantiomeric eicosanoids in human whole blood. Combining chiral chromatography with HRMS, we detected differences in the variety of chiral species and the magnitude of response during in vitro activation of inflammatory versus coagulation pathways.

It is important to be able to distinguish the enantiomeric excess of the isomeric compounds with high specificity and sensitivity to segregate an enzymatic process (chiral products) from oxidative stress (racemic products). In a biological system, which has the enzymatic machinery on the one hand, and is prone to oxidative stress, on the other hand, both enzymatic and nonenzymatic oxidations will contribute to enantiomer formation to varied degrees. Typically, resolution of such molecules has been conducted using normal-phase chiral chromatography coupled with UV detection (13) or MS (14). Unfortunately, the sensitivity of MS analysis using conventional ESI methodology and normal-phase solvents is rather poor. This methodology has a quite limited applicability for analyzing nonesterified bioactive lipids such as 15-HETE. Sensitivity can be enhanced by the use of APCI-MS after derivatization with an electron-capturing group like PFB. This technique, called ECAPCI-MS, provides a substantial increase in sensitivity when compared with conventional negative ion APCI methodology of underivatized analytes (15). We reasoned that EC methodology could provide an excellent way to analyze chiral lipids by HRMS, analogous to using low-resolution LC-MS/MS (16).

Previous studies showed that PFB derivatization increases sensitivity of detection for carboxy- (17) or phenol-containing (18) molecules. These studies used triple quadrupole instruments with unit resolution in MRM modes. The MS/HRMS spectra for each isomeric HETE showed specific fragments, with the α-cleavage next to the hydroxyl group being the most intense fragment, as reported before for methods involving triple quadrupole instruments (11, 19, 20).

We used only one HETE internal standard {[^2^H_8_]15(*S*)-HETE} in this report. However, deuterated internal standards are available for almost all the HETEs, and their usage will increase the accuracy and precision of the method. More rigorous analysis can be achieved by including additional heavy isotope internal standards into the sample preparation step, both for less polar eicosanoids, such as the oxo-ETEs, and for more polar products, such as the prostaglandins and leukotrienes. An extended targeted lipidomics method could also include metabolites from other pathways, like epoxyeicosatrienoic acids that result from the cytochrome P450 pathway (21).

Comparison of serum to untreated plasma revealed a robust production of chiral species during blood clotting. Anionic surfaces trigger the intrinsic pathway of coagulation through the auto-activation of the contact system factor XII (FXII) freely circulating in the bloodstream (22, 23). In this study, we activated blood clotting in vitro using negatively charged glassware, and measured the maximum capacity of human blood to synthesize bioactive eicosanoids, particularly chiral HETEs, during coagulation. The 12(*S*)-HETE, 12(*S*)-HHT, and 15(*S*)-HETE were the most abundant hydroxylated chiral lipids in serum, which, together with TxB_2_, are attributable to platelet activation during coagulation (22, 24, 25). Platelets produce eicosanoids through the oxidation of arachidonic acid by 12-LOX and COX-1 enzymes, and nonenzymatic conversion of the PGH_2_ substrate (25). Serum 12(*S*)-HETE is exclusively formed by the platelet-type, 12(*S*)-LOX (1, 26), while 12(*R*)-HHT is generated nonenzymatically and through the enzymatic conversion of arachidonic acid by COX-1 and Tx synthase (25). Considering that 12(*R*)-HETE is synthesized by cytochrome P450 in the ocular system (27) and by 12(*R*)-LOX in the epidermis and tonsils (28, 29), we hypothesize that low levels of serum 12(*R*)-HETE are formed nonenzymatically. The role of 12(*S*)-HETE in platelet biology is not fully understood, with studies reporting both activation (30–32) and inhibition (33, 34) of platelet aggregation. The 12-HETE has been implicated in the pathophysiology of cancer, arteriosclerosis, and diabetes (26). The 12(*S*)-HHT was formed in comparable amounts to TxB_2_ and, overall, accounts for roughly 30% of the arachidonate metabolites formed by activated platelets (35). Despite considerable production of 12(*S*)-HHT during blood clotting, its biological function is still unknown. Sources of serum 15(*S*)-HETE include eosinophil 15-LOX-1 and both COX isoforms, COX-1 from platelets and COX-2 from monocytes (1, 14, 36). However, 15(*S*)-HETE, as well as the *R*-enantiomer, originated primarily from platelet COX-1. The 15-HETE exerts pro-coagulant effects by increasing thrombin-induced activation and aggregation of platelets (34). Serum 11(*R*)-HETE can also be attributable to platelet COX-1 that generates 11-HETE only in the *R*-configuration (2). Finally, production of 5(*S*)-HETE through catalysis by 5-LOX indicates activation of neutrophils, which contribute to coagulation and thrombosis (37, 38). Taken together, our method detected a wide variety of chiral HETEs in coagulated blood and confirmed the predominance of products formed enzymatically over those formed nonenzymatically.

As previously reported (6), LPS and zymosan differed in their kinetics of maximum response and the predominant lipid products formed. LPS elevated 12(*S*)-HHT, 15(*S*)-HETE, and 11(*R*)-HETE. Plasma 15(*S*)-HETE is synthesized by 15-LOX-1, expressed in eosinophils, monocytes, reticulocytes, and by COX isoforms (1, 36). LPS potentiated 15(*R*)-HETE only at a later time point, consistent with its delayed induction of COX-2 (2). Upregulation of 11(*R*)-HETE by LPS can also be attributable to COX-2 induction (2). In contrast, zymosan stimulated production of most studied HETEs and 12(*S*)-HHT upon short-term incubation, with neutrophil 5-LOX-derived 5(*S*)-HETE as a dominant product. The increase in 11(*R*)-HETE, 12(*S*)-HHT, and 15(*S*)-HETE is attributable to the zymosan induction of COX-2 in neutrophils and possibly other blood cell types (39, 40). Upregulation of nonenzymatic species, such as 5(*R*)-HETE, 8(*R*)- and 8(*S*)-HETEs, 9(*R*)- and 9(*S*)-HETEs, 11(*S*)-HETE, 12(*R*)-HETE, and 15(*R*)-HETE, may be due to oxidative stress triggered by in vitro stimulation of whole blood (41–45).

Overall, using chiral chromatography and HRMS, we detected a much broader variety of chiral lipids than reported previously (6). The 12(*S*)-HETE was robustly and continuously produced in human whole blood, irrespective of the stimulating conditions. This is consistent with the observation that platelet 12(*S*)-LOX, unlike COX-1, continues to oxidize arachidonic acid over time, and that these enzymes use different pools of arachidonic acid and differentially rely on cytosolic phospholipase A_2_ to release the arachidonate substrate (46). The 20-HETE is a potent vasoconstrictor made by vascular smooth muscle cells, among other cellular sources (2). In whole blood, it can be generated by stimulated neutrophils (47). Interestingly, plasma 20-HETE decreased after a long-term incubation with either LPS or zymosan. Activated platelets metabolize 20-HETE via 12(*S*)-LOX- and COX-1-dependent mechanisms (48) that may account for reduction in 20-HETE levels in stimulated whole blood. In addition, a decrease in 20-HETE may result from esterification into phospholipids of cellular membranes (49).

In conclusion, we have developed a method that allows for a rapid quantification with high sensitivity and specificity of enantiomeric lipids, particularly HETEs, in human whole blood. Although the precise origin of each chiral product is speculative, the method differentiates enzymatic versus nonenzymatic chiral species. This analytical method can also be readily applied to the interrogation of other classes of nonesterified oxidized lipids in various biological matrices (49).
